# The first activation study of a δ-carbonic anhydrase: TweCAδ from the diatom *Thalassiosira weissflogii* is effectively activated by amines and amino acids

**DOI:** 10.1080/14756366.2018.1447570

**Published:** 2018-03-14

**Authors:** Andrea Angeli, Fatmah A. S. Alasmary, Sonia Del Prete, Sameh M. Osman, Zeid AlOthman, William A. Donald, Clemente Capasso, Claudiu T. Supuran

**Affiliations:** a Department of Neurofarba, Sezione di Scienze Farmaceutiche e Nutraceutiche, Università degli Studi di Firenze, Florence, Italy;; b Department of Chemistry, College of Science, King Saud University, Riyadh, Saudi Arabia;; c Istituto di Bioscienze e Biorisorse, CNR, Napoli, Italy;; d School of Chemistry, University of New South Wales, Sydney, Australia

**Keywords:** Carbonic anhydrase, metalloenzymes, diatoms, activators, *Thalassiosira weissflogii*

## Abstract

The activation of the δ-class carbonic anhydrase (CAs, EC 4.2.1.1) from the diatom *Thalassiosira weissflogii* (TweCAδ) was investigated using a panel of natural and non-natural amino acids and amines. The most effective activator of TweCAδ was d-Tyr (K_A_ of 51 nM), whereas several other amino acids and amines, such as L-His, L-Trp, d-Trp, dopamine and serotonin were submicromolar activators (K_A_s from 0.51 to 0.93 µM). The most ineffective activator of TweCAδ was 4-amino-l-Phe (18.9 µM), whereas d-His, l-/d-Phe, l-/d-DOPA, l-Tyr, histamine, some pyridyl-alkylamines, l-adrenaline and aminoethyl-piperazine/morpholine were moderately potent activators (K_A_s from 1.34 to 8.16 µM). For any δ-CA, there are no data on the crystal structure, homology modelling and the amino acid residues that are responsible for proton transfer to the active site are currently unknown making it challenging to provide a detailed rational for these findings. However, these data provide further evidence that this class of underexplored CA deserves more attention.

## 1. Introduction

Carbonic anhydrases are an ubiquitous family of enzymes that catalyse the rapid interconversion between CO_2_ and water to bicarbonate and protons. Of the seven genetically distinct families of CA enzymes known to date, the δ-class carbonic anhydrases (CAs, EC 4.2.1.1) are the least investigated. In 2000, Morel’s group discovered δ-CAs in the diatom *Thalassiosira weissflogii*^1^, which was initially denominated TWCA1. Subsequently, a number of orthologues of this specific enzymes have been identified in most diatoms from natural phytoplankton assemblages and are responsible (along with other CAs) for CO_2_ fixation by marine organisms^2^. A related species of the original diatom in which these enzymes were reported, *T. pseudonana*, was demonstrated to possess genes for three α-, five γ-, four δ- and one ζ-CAs^3^. However, none of these enzymes have been cloned and characterised in detail to date. Thus, diatoms can be considered the organisms with the most intricate and poorly understood distribution of CAs, and the roles of CAs are far from being well understood with the exception of their important role in CO_2_ fixation and photosynthesis, as they provide bicarbonate or CO_2_ to ribulose-1, 5-bisphosphate carboxylase/oxygenase (RUBISCO)^1–3^.

In 2013, Lee et al.^4^ cloned and purified the δ-CA from *T. weissflogii* and investigated its esterase activity (and not its CO_2_ hydrase activity) using the substrate, 4-nitrophenyl acetate. Our group demonstrated that such esterase activity is artefactual^5^; i.e. the activity does not result from hydrolysis of the ester at the zinc hydroxide active site of the enzyme. This was confirmed by performing the esterase hydrolysis catalysed reaction in the presence of the enzyme with and without a potent CA inhibitor (CAI) that selectively binds to the zinc active site^5^. This highlights the importance of performing control experiments to confirm CA enzymatic activity. Our group characterised the CO_2_ hydrase activity of this enzyme (denominated by us TweCAδ^5^) and reported the first anion and sulphonamide inhibition studies for any δ-class enzyme. These data demonstrate that TweCAδ is similar to other CAs belonging to the α-, β-, γ-, ζ-, η- and θ-CAs^6–8^; i.e. TweCA is an excellent catalyst for the hydration of CO_2_ to bicarbonate and hydronium ions, and that its activity may be inhibited by anions and sulphonamides, the two main classes of simple CAIs^5–8^. However, no activation studies of this enzyme have been reported to date, although the CA activators (CAAs) are an important class of modulators for the activity of CA enzymes^9^^,^^10^.

CAAs have been demonstrated to participate in the CA catalytic cycle^9^, which is shown schematically in the following equations:

(1) $$\begin{array}{c} {\text{H}}_{\text{2}}\text{O}\\ {\text{EZn}}^{\text{2}+}—{\text{OH}}^{-}+{\text{CO}}_{\text{2}}\Leftrightarrow {\text{EZn}}^{\text{2}+}–{\text{HCO}}_{{\text{3}}^{\text{-}}}\Leftrightarrow {\text{EZn}}^{\text{2}+}—{\text{OH}}_{\text{2}}+{\text{HCO}}_{\text{3}}^{\text{-}} \end{array}$$

(2) $${\text{EZn}}^{\text{2}+}—{\text{OH}}_{\text{2}}\Leftrightarrow {\text{EZn}}^{\text{2}+}—{\text{HO}}^{-}+{\text{ H}}^{+}$$

In the first step, a zinc-bound hydroxide species of the enzyme nucleophilically attacks the CO_2_ substrate, which is bound in a hydrophobic pocket nearby and is optimally orientated for the hydration reaction to occurs (Equation (1))^7–9^. The second part of the process involves the replacement of bicarbonate formed in the hydration reaction by an incoming water molecule to form the acidic enzymatic species, EZn^2+^–OH_2_ (Equation (1)). In order to regenerate the zinc hydroxide species, a proton is transferred from the Zn(II)-bound water molecule to the external medium (Equation (2)), which is the rate-determining step of the entire catalytic cycle^7–9^:

(3) $$\begin{array}{c} {\text{EZn}}^{\text{2}+}—{\text{OH}}_{\text{2}}+\text{A}\Leftrightarrow \left[{\text{EZn}}^{\text{2}+}—{\text{OH}}_{\text{2}}-\text{A}\right]\Leftrightarrow \left[{\text{EZn}}^{\text{2}+}—{\text{HO}}^{-}-{\text{AH}}^{+}\right]\Leftrightarrow {\text{EZn}}^{\text{2}+}—{\text{HO}}^{-}+{\text{AH}}^{+}\\ \text{enzyme }–\text{ activator complexes} \end{array}$$

In the presence of activators (A in Equation (3)), this rate-determining step is facilitated by an additional proton release pathway, which involves the activator A bound within the enzyme active site. By forming an enzyme-activator complex, the proton transfer reaction becomes intramolecular and thus more rapid compared to the intermolecular process in which for example buffers can take part^7–10^. The enzyme–activator complexes were thoroughly characterised for α-CAs of human (h) origin, such as hCA I and II, by means of kinetic and X-ray crystallographic techniques, which allowed the activator-binding site within the CA cavity and the structure–activity relationship governing these processes to be determined^9^^,^^10^. However, CAA research has been relatively neglected compared with that for CAI. Inhibitors of the sulphonamide type^11–13^ that target CAs belonging to various classes and from various organisms have been extensively studied, and possess clinical applications as drugs for the treatment of oedema, glaucoma, epilepsy, obesity and cancer^14^^,^^15^. Recently, CAIs were also proposed as an alternative for the management of neuropathic pain^16^, cerebral ischemia^16^, arthritis^17^ and potentially as anti-infectives^18^. In contrast, the activation of CAs by naturally occurring amines and amino acids may play a role in increasing the activity of CAs in pathogens^19^. In this paper, we report the first activation study of a δ-CA, investigating the activation profile with amines and amino acids of TweCAδ.

## 2. Materials and methods

### 2.1. Materials

Amino acids and amines **1–19** were commercially available, highest purity reagents from Sigma-Aldrich, Milan, Italy. TweCAδ was a recombinant protein produced as reported earlier by our group^5^.

### 2.2. CA enzyme activation assay

An Sx.18Mv-R Applied Photophysics (Oxford, UK) stopped-flow instrument has been used to assay the catalytic activity of various CA isozymes for CO_2_ hydration reaction^18^. Phenol red (at a concentration of 0.2 mM) was used as indicator, working at the absorbance maximum of 557 nm, with 10 mM Hepes (pH 7.5) as buffer, and 0.1 M Na_2_SO_4_ (for maintaining constant ionic strength, which is not inhibitory against TweCAδ^5^), following the CA-catalyzed CO_2_ hydration reaction for a period of 10 s at 25 °C. The CO_2_ concentrations ranged from 1.7 to 17 mM for the determination of the kinetic parameters and activation constants. For each activator, at least six traces of the initial 5–10% of the reaction have been used for determining the initial velocity. The uncatalyzed rates were determined in the same manner and subtracted from the total observed rates. Stock solutions of activators (10 mM) were prepared in distilled–deionized water and dilutions up to 1 nM were done thereafter with the assay buffer. Activator and enzyme solutions were pre-incubated together for 15 min (standard assay at room temperature) prior to assay, in order to allow for the formation of the E–A complex. The activation constant (K_A_), defined similarly with the inhibition constant K_I_, can be obtained by considering the classical Michaelis–Menten equation (Equation (4)), which has been fitted by non-linear least squares by using PRISM 3:

(4) $$\text{v }={\text{v}}_{\text{max}}/\left\{\text{1}+{\text{K}}_{\text{M}}/\left[\text{S}\right]\left(\text{1}+{\left[\text{A}\right]}_{\text{f}}/{\text{K}}_{\text{A}}\right)\right\}$$

where [A]_f_ is the free concentration of activator.

Working at substrate concentrations considerably lower than K_M_ ([S] **≪**K_M_), and considering that [A]_f_ can be represented in the form of the total concentration of the enzyme ([E]_t_) and activator ([A]_t_), the obtained competitive steady-state equation for determining the activation constant is given by the following equation:

(5) $$\text{v}={\text{v}}_{0}.{\text{K}}_{\text{A}}/\{{\text{K}}_{\text{A}}+{\left({\left[\text{A}\right]}_{\text{t}}-0.\text{5}\{\left({\left[\text{A}\right]}_{\text{t}}+{\left[\text{E}\right]}_{\text{t}}+{\text{K}}_{\text{A}}\right)-{\left({\left[\text{A}\right]}_{\text{t}}+{\left[\text{E}\right]}_{\text{t}}+{\text{K}}_{\text{A}}\right)}^{\text{2}}-\text{4}{\left[\text{A}\right]}_{\text{t}}.{\left[\text{E}\right]}_{\text{t}}\right)}^{\text{1}/\text{2}}\}\}$$

where v_0_ represents the initial velocity of the enzyme-catalyzed reaction in the absence of activator^19–27^.

## 3. Results and discussion

**Figure 1.**
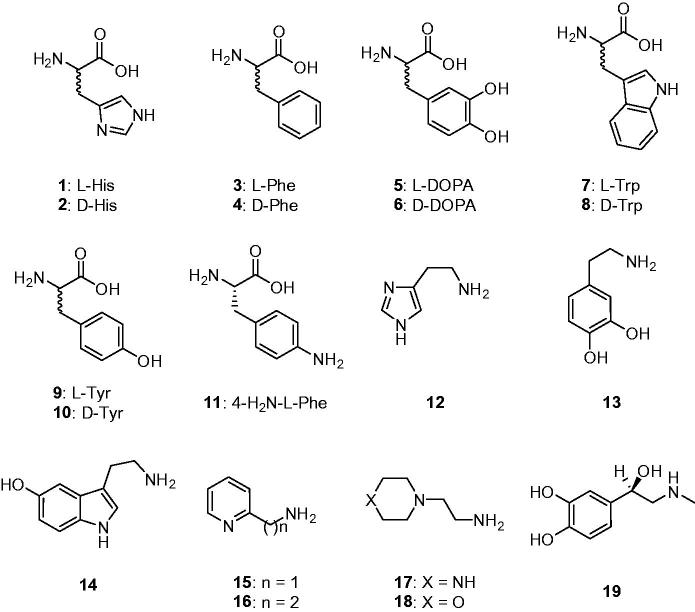
Amino acids **1–11** and amines **12–19** investigated as TweCAδ activators.

Natural and non-natural amino acids and amines **1–19** were included among the investigated compounds as activators of TweCAδ (Figure 1). These compounds were employed for investigations as CAAs against many classes of CAs, including the bacterial, archaeal and mammalian ones mentioned earlier^10^^,^^11^^,^^21–29^. The presence of protonatable moieties of the amine, carboxylate or imidazole type present in these derivatives makes them appropriate for participating in the proton shuttling processes between the active site and the reaction medium, as described by Equation (3).

Data of Table 1 show that TweCAδ shows a CO_2_ hydrase activity quite similar to hCA I, a widely spread isoform in humans^8^. Both the first-order kinetic constant (*k*_cat_) and the K_M_ of the two enzymes are very similar. In the presence of 10 µM l-Trp as activator, the K_M_ of TweCAδ remained unchanged (data not shown) but the *k*_cat_ was 8.15 times higher than in the absence of the activator (Table 1). This situation has been observed for all CAs investigated to date, belonging to all known CA genetic families, proving that presumably the CA activation mechanism is similar for all enzyme classes, involving facilitation of the proton transfer process by the activator molecule bound within the enzyme active site in the enzyme–activator complex.

**Table 1.** Activation of human carbonic anhydrase (hCA) isozymes I, II, and TweCAδ with l-Trp, at 25 °C, for the CO_2_ hydration reaction^20^.

Isozyme	*k*_cat_^a^	K_M_^a^	(*k*_cat_) _l-Trp_^b^	K_A_^c^ (μM)
	(s^−1^)	(mM)	(s^−1^)	l-Trp
hCA I^d^	2.0 × 10^5^	4.0	3.4 × 10^5^	44
hCA II^d^	1.4 × 10^6^	9.3	4.9 × 10^6^	27
TweCAδ^e^	1.3 × 10^5^	3.9	10.6 × 10^5^	0.93

a Observed catalytic rate without activator. K_M_ values in the presence and the absence of activators were the same for the various CAs (data not shown).

b Observed catalytic rate in the presence of 10 μM activator.

c The activation constant (K_A_) for each enzyme was obtained by fitting the observed catalytic enhancements as a function of the activator concentration^21–27^. Mean from at least three determinations by a stopped-flow, CO_2_ hydrase method^20^. Standard errors were in the range of 5–10% of the reported values (data not shown).

d Human recombinant isozymes, from Ref.^26^.

e Diatom recombinant enzyme, this work.

Data of Table 2 show the TweCAδ activation with amino acids and amines **1–19**. The activation profile with the same compounds for the widespread, physiologically relevant isoforms hCA I and II (belonging to the α-CA family) are also shown for comparison reasons. The following structure-activity relationship can be inferred for TweCAδ activation with these compounds: (i) the most effective TweCAδ activator was d-Tyr, with an activation constant of 51 nM, whereas several other amino acids and amines, such as l-His, l-Trp, d-Trp, dopamine and serotonin were submicromolar activators, with K_A_s ranging between 0.51 and 0.93 µM; (ii) the most ineffective activator of TweCAδ was 4-amino-l-Phe, with an activation constant of 18.9 µM; (iii) the remaining derivatives investigated were effective to moderately potent activators, with K_A_s ranging between 1.34 and 8.16 µM. Thus, the SAR for these compounds is rather “flat” because most were rather effective activators of this enzyme. However, some features will be discussed. The stereochemistry of the chiral centre for the amino acid derivatives seems to not be very important, since both l- (e.g. l-His, l-Trp) and d-amino acid derivatives (e.g. d-Trp, d-Tyr) showed effective TweCAδ activation (Table 2). Small changes in the scaffold of an activator led to important differences of activity. For example, introduction of an amino moiety in the 4 position of the phenyl ring in l-Phe (a rather effective activator) let to a massive loss of efficacy in compound **11**, which was 8.8 times a less efficient activator compared with **3**. The loss of the carboxyl moiety from l- or d-DOPA led to enhanced activating properties in dopamine **13**, compared with **5** and **6**. Another example is the nature of the X moiety in **17** and **18**, with the morpholine **18** being 1.7 times a weaker activator compared to the piperazine **17**. (iv) The activation profile of the δ-class enzyme investigated here is very different from that of the α-CAs hCA I and II. Since no crystal structure (or even modelling) of any δ-CA is available so far, it is challenging to rationalize in detail these data. In addition, the proton transfer residue(s) responsible for shuttling protons to and from the active site in this class of CAs is unknown.

**Table 2.** Activation constants of hCA I, hCA II and the bacterial TweCAδ with amino acids and amines **1–19**, by a stopped-flow CO_2_ hydrase assay^20^.

		K_A_ (μM)^a^		
No.	Compound	hCA I^b^	hCA II^b^	TweCAδ^c^
**1**	l-His	0.03	10.9	0.75
**2**	d-His	0.09	43	4.90
**3**	l-Phe	0.07	0.013	2.15
**4**	d-Phe	86	0.035	1.96
**5**	l-DOPA	3.1	11.4	2.11
**6**	d-DOPA	4.9	7.8	6.24
**7**	l-Trp	44	27	0.93
**8**	d-Trp	41	12	0.69
**9**	l-Tyr	0.02	0.011	1.52
**10**	d-Tyr	0.04	0.013	0.051
**11**	4-H_2_N-l-Phe	0.24	0.15	18.9
**12**	Histamine	2.1	125	1.34
**13**	Dopamine	13.5	9.20	0.51
**14**	Serotonin	45	50	0.90
**15**	2-Pyridyl-methylamine	26	34	5.28
**16**	2-(2-Aminoethyl)pyridine	13	15	8.16
**17**	1-(2-Aminoethyl)-piperazine	7.4	2.30	4.37
**18**	4-(2-Aminoethyl)-morpholine 0.14	0.19	7.39	
**19**	l-Adrenaline	0.09	96	2.43

a Mean from three determinations by a stopped-flow, CO_2_ hydrase method^20^. Standard errors were in the range of 5–10% of the reported values (data not shown).

b Human recombinant isozymes, stopped flow CO_2_ hydrase assay method^26^.

c Diatom enzyme, this work.

## 4. Conclusions

The first activation study of a δ-class CA is reported. The most effective TweCAδ activator was d-Tyr, with an activation constant of 51 nM, whereas several other amino acids and amines, such as l-His, l-Trp, d-Trp, dopamine and serotonin were submicromolar activators, with K_A_s ranging between 0.51 and 0.93 µM. The most ineffective TweCAδ activator was 4-amino-l-Phe, with an activation constant of 18.9 µM, whereas d-His, l-/d-Phe, l-/d-DOPA, l-Tyr, histamine, some pyridyl-alkylamines, l-adrenaline and aminoethyl-piperazine/morpholine were somewhat potent activators, with K_A_s ranging between 1.34 and 8.16 µM. Since no crystal structure or homology modelling of any δ-CA is available so far, it is challenging to rationalize in detail our findings. In the future, crystallography, homology modelling and mutagenesis studies are likely to provide valuable mechanistic details into the role of CAAs in this relatively new class of CA. This may lead to a more complete understanding of the role of nature amines and amino acids in the modulation of CO_2_ fixation in phytoplankton.
